# Interaction of designed cationic antimicrobial peptides with the outer membrane of gram-negative bacteria

**DOI:** 10.1038/s41598-024-51716-1

**Published:** 2024-01-22

**Authors:** Shelley He, Charles M. Deber

**Affiliations:** 1 Program in Molecular Medicine, Research Institute, Hospital for Sick Children, Toronto, ON M5G 0A4 Canada; 2 Department of Biochemistry, University of Toronto, Toronto, M5S 1A8 Canada

**Keywords:** Biophysical chemistry, Peptides

## Abstract

The outer membrane (OM) is a hallmark feature of gram-negative bacteria that provides the species with heightened resistance against antibiotic threats while cationic antimicrobial peptides (CAPs) are natural antibiotics broadly recognized for their ability to disrupt bacterial membranes. It has been well-established that lipopolysaccharides present on the OM are among major targets of CAP activity against gram-negative species. Here we investigate how the relative distribution of charged residues along the primary peptide sequence, in conjunction with its overall hydrophobicity, affects such peptide-OM interactions in the natural CAP Ponericin W1. Using a designed peptide library derived from Ponericin W1, we determined that the consecutive placement of Lys residues at the peptide N- or C-terminus (ex. “PonN”: KKKKKKWLGSALIGALLPSVVGLFQ) enhances peptide binding affinity to OM lipopolysaccharides compared to constructs where Lys residues are interspersed throughout the primary sequence (ex. “PonAmp”: WLKKALKIGAKLLPSVVKLFKGSGQ). Antimicrobial activity against multidrug resistant strains of *Pseudomonas aeruginosa* was similarly found to be highest among Lys-clustered sequences. Our findings suggest that while native Ponericin W1 exerts its initial activity at the OM, Lys-clustering may be a promising means to enhance potency towards this interface, thereby augmenting peptide entry and activity at the IM, with apparent advantage against multidrug-resistant species.

## Introduction

Cationic antimicrobial peptides (CAPs) are natural broad-spectrum antibiotics produced by all living organisms^1,2^. They are typically characterized as short, hydrophobic molecules that harbor a net positive charge^3^. In the last decade, CAPs have gained therapeutic attention due to their rapid and direct mode of action at bacterial cell membranes^4–8^. This property of CAPs offers a functional advantage over traditional small molecule drugs that generally target specific intracellular sites with relatively high susceptibility to bacterial resistance^9–14^.

Many sequence-to-function studies of CAPs prioritize their activity against artificial membranes mimicking the bacterial cytoplasmic inner membrane (IM)—a phospholipid bilayer predominantly composed of zwitterionic phosphatidylethanolamine (PE) and anionic phosphatidylglycerol (PG)^2,15,16^. As such, there are numerous investigations that characterize the biophysical details of CAP interaction with model bilayer systems in vitro^17^. While there is a rich diversity in experimental design, it is largely agreed upon that at physiological pH, PG lipids on the outer leaflet of the bilayer provide the surface with a net negative charge, which is used by CAPs as an electrostatic anchor^2,17^. Upon arrival at the IM, most CAPs adopt amphipathic secondary structures (viz. one structural motif lined by hydrophobic residues while another is lined by charged residues) that can assemble to form transmembrane pores or accumulate in a carpeted manner at the bilayer surface, leading to tension and cellular collapse^18–20^.

While the details of CAP activity at the IM have been extensively studied, an equally important sequence in this pathway pertains to the route of permeation taken by peptides across the outer membrane (OM) to reach the IM. The OM is an evolutionary advantage of gram-negative bacteria that provides the species with enhanced robustness against harsh extracellular environments^21–23^. It has been proposed that the development of this secondary membrane may have originated in response to selective pressures from early antibiotic-producing organisms in nature^21,24–26^. Interestingly, the OM does not solely function as a physical barrier, but also a chemical one by virtue of LPS toxicity (broadly known as endotoxicity) to the affected host^27^. In this regard, we note the remarkable persistence of gram-positive species despite their comparatively vulnerable biology. The co-existence of gram-negative and gram-positive bacteria nevertheless emphasizes the optimized design of the cell envelope (including the membranes and any other structural components surrounding the cytoplasm)^28^. In an evolutionary dialogue, it can be thought that the development of a highly refined bacterial cell envelope has been counterbalanced in nature per se by CAPs that are capable of targeting cell membranes with broad-spectrum specificity.

The OM is thus a selective barrier unique to gram-negative bacteria^29–31^. Some CAPs (ex. polymyxins) primarily target the OM, enabling subsequent IM disruption only because this initial threshold is compromised^32,33^. Structurally, the OM is an asymmetric bilayer with phospholipids confined to the inner leaflet and complex glycolipids, termed lipopolysaccharides (LPS), forming the outer leaflet^29,31^. LPS are large, polyanionic molecules comprised of a lipid base (lipid A) linked to an extended chain of sugar moieties (including a core oligosaccharide commonly composed of Kdo residues, heptose, and hexose, and a variable O-antigen moiety)^27^. The amphipathic nature and dense lateral organization of LPS facilitated by highly saturated acyl chains are primarily responsible for hindering the passage of a wide range of molecules, including antibiotics^34^. Small hydrophobic drugs (ex. macrolides) are capable of diffusing across the OM while hydrophilic species (ex. β-lactams) generally utilize protein channels (porins)^29^. In this regard, the ability of CAPs—comparatively large and amphipathic molecules—to permeate the OM independent of diffusion or porins underscores a delicate chemical balance. Indeed, the course taken by peptides across the OM to reach the IM has been a subject of increasing interest and it has been reported that chemical alterations (ex., charge neutralization to lipid A granted by the removal or modification of phosphate groups) to LPS constituents of the bacterial OM are among principal mechanisms of gram-negative resistance to CAPs^35^.

In light of the importance of CAP-OM interactions towards antimicrobial function, the present work investigates how charged residue placement (i.e., along the primary peptide sequence) and overall hydrophobicity influence the interaction of Ponericin W1 (WLGSALKIGAKLLPSVVGLFKKKKQ, a natural CAP isolated from the venom of *Pachycondyla goeldii* ants^36^) with models of the bacterial OM. Modified analogs of Ponericin W1 have been previously shown to inhibit bacterial growth by interacting with, and promoting leakage at the IM^37^. The nature of peptide-IM interactions (i.e., peptide secondary structuring, depth of insertion) was found to vary depending on Lys positioning within the primary peptide sequence^37^. Our findings herein glean further insight into the sequence-to-function relationship of the Ponericin W1 library at the OM interface. Notably, we report that the consecutive placement (or “clustering”) of six Lys residues at the peptide N- or C-terminus offers enhanced OM binding compared to constructs where Lys residues are interspersed throughout the primary sequence, as they are commonly positioned in nature. As well, peptides featuring a Lys-clustered archetype consistently exhibit the greatest antimicrobial activity against multidrug resistant (MDR) strains of *Pseudomonas aeruginosa,* likely in part due to their heightened efficiency at crossing the OM.

## Results

### Peptide library

The present work builds on an existing peptide library derived from Ponericin W1, a natural CAP isolated from the venom of *Pachycondyla goeldii* ants (Table 1). Firstly, peptide analogues of Ponericin W1 were designed based on the systematic rearrangement of Lys residues within the primary peptide sequence such that they were interspersed along the sequence length to provide a maximal amphipathic helix (‘PonAmp’; degree of helix amphipathicity measured based on calculations of hydrophobic moment^38,39^). Importantly, here we have defined amphipathicity on the basis of a presumed helical structure (viz. one helical face is predominantly charged while the opposing helical face is predominantly hydrophobic). Thus, the peptides we have designated as the PonAmp group in Table 1 are referred to as “helical amphipathic”. While helical amphipathicity is well-recognized as a pre-requisite for CAP activity at membrane interfaces^1^, “linear amphipathicity”, where charged residues are clustered toward one of the peptide termini, has also been reported to relay a critical role towards the membrane activity of some synthetic CAPs^40^. Accordingly, in the present work, the sets of peptides designated ‘PonN’ and ‘PonC’ in Table 1 contain all their Lys residues clustered respectively at the N- or C-terminus, resulting in an accompanying uninterrupted hydrophobic sequence^37^; as such, these peptides have linear amphipathicity. We note that native Ponericin W1 partially displays linear amphipathicity by harboring a consecutive sequence of four Lys residues close to the C-terminus. Interestingly, the composition of these linear amphipathic peptides is analogous to the chemical arrangement of highly membrane-active detergents, which are characterized by a polar head group linked to a hydrophobic hydrocarbon-like tail^41^.

**Table 1:**
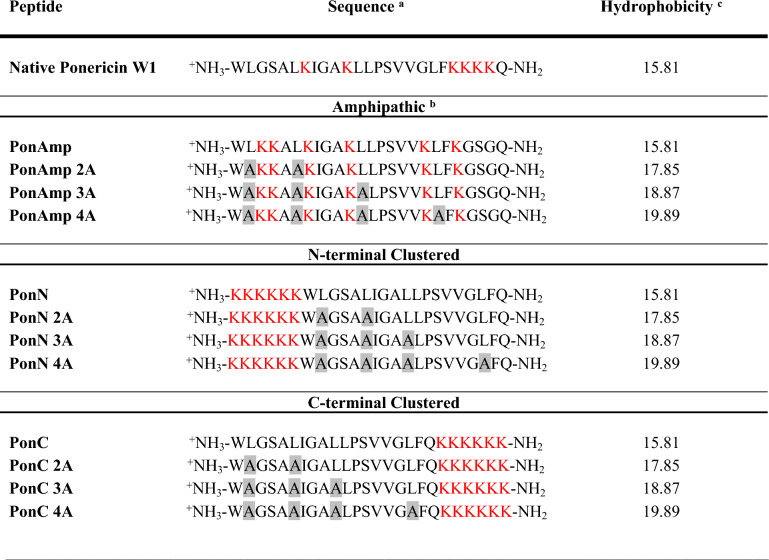
Sequences of native Ponericin W1 and its helical amphipathic and Lys-clustered variants. ^a^Lys residues are rendered in red. Stepwise Leu-to-Ala substitutions are indicated in highlighted gray boxes. ^b^Nomenclature for PonAmp refers to the helical amphipathicity of the designed peptides. ^c^Hydrophobicity measured on the Octanol-Interface scale as ΔG (kcal/mol), calculated for partitioning from water to bilayer where a greater value indicates a less hydrophobic sequence^43^.

In designing the Ponericin peptide library, our principal goal is to gauge systematically how the relative distribution of charged residues influences peptide-membrane interactions. As well, hydrophobicity is of particular interest to our investigations as earlier studies on the Ponericin W1 library reveal the highly toxic nature of native Ponericin W1 against mammalian red blood cells^37^. Thus, stepwise Leu-to-Ala substitutions (up to four substitutions) were subsequently applied to each parent analog to simultaneously evaluate the contribution of overall sequence hydrophobicity. All peptides in our designed library carry a + 7 charge at physiological pH (containing six Lys residues and a free amino end at the N-terminus) while in principle retaining sufficient hydrophobic character as required for membrane insertion^42^. Through balancing measures of host safety and antimicrobial efficacy, we found that a minimum of two Leu-to-Ala substitutions is able to universally eliminate mammalian host cell toxicity up to 50 μM, without jeopardizing activity at the IM^37^. As such, we were able to design sets of peptides with measurably improved therapeutic potential compared to native Ponericin W1.

### Lys-clustered peptides exhibit potent antimicrobial activity against the gram-negative bacterium, *Pseudomonas aeruginosa*

**Figure 1:**
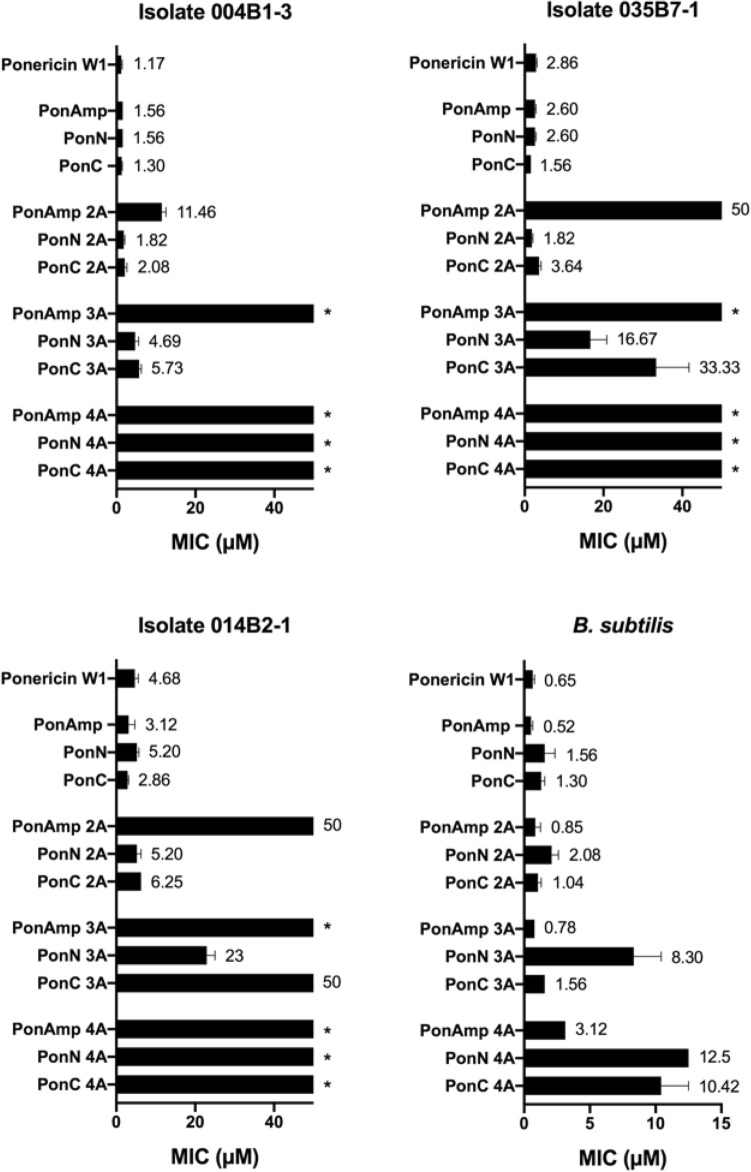
Minimum inhibitory concentrations (MICs) for the present peptide library against three clinical isolates of MDR *P. aeruginosa* and a laboratory strain of *B. subtilis.* Increasing concentrations of peptides were independently incubated with bacterial cells for 20 h. MIC was calculated based on bacterial growth in an untreated control and in 1% Triton. Data represent the average from n = 6 replicates from N = 3 biological experiments performed on different days; error bars depict the standard error of the mean. Asterisk denotes MIC values beyond the highest tested concentration (50 μM).

We began our investigations by testing the antimicrobial efficacy of our peptide library against *P. aeruginosa*, an opportunistic gram-negative bacterium found in patients with cystic fibrosis (CF)^44,45^. MDR isolates of *P. aeruginosa* obtained from chronically infected CF patients were independently challenged with the peptide library using a minimum inhibitory concentration assay (MIC, defined as the lowest drug concentration required to inhibit bacterial growth). Native Ponericin W1, as well as all derived parent sequences consistently demonstrated high levels of inhibitory function with low MIC values nearing 1 μM (Fig. 1). The incorporation of two Leu-to-Ala substitutions measurably impedes activity of the helical amphipathic construct PonAmp 2A (MIC rising to 50 μM), however does not significantly impact the activity of Lys clustered PonN 2A (average MIC ~ 3 μM) or PonC 2A (average MIC ~ 4 μM). A similar trend is observed among the 3A analogs. At four Leu-to-Ala substitutions, activity is universally lost as the sequences no longer meet the minimum hydrophobicity threshold for spontaneous membrane insertion^42^.

To compare antimicrobial function in a species lacking the OM, we complemented this dataset with evaluations of MIC against a common gram-positive model, *Bacillus subtilis* (Fig. 1). In general, *B. subtilis* is a sensitive strain with greater susceptibility to peptide activity (MIC < 15 μM across all sequence analogs) than *P. aeruginosa.* Intriguingly, in the absence of an OM, Lys-clustered sequences now exhibit greater losses in antimicrobial potential with increasing Leu-to-Ala substitutions compared to their helical amphipathic counterpart.

### Lys-clustered peptides bind LPS with high affinity

**Figure 2:**
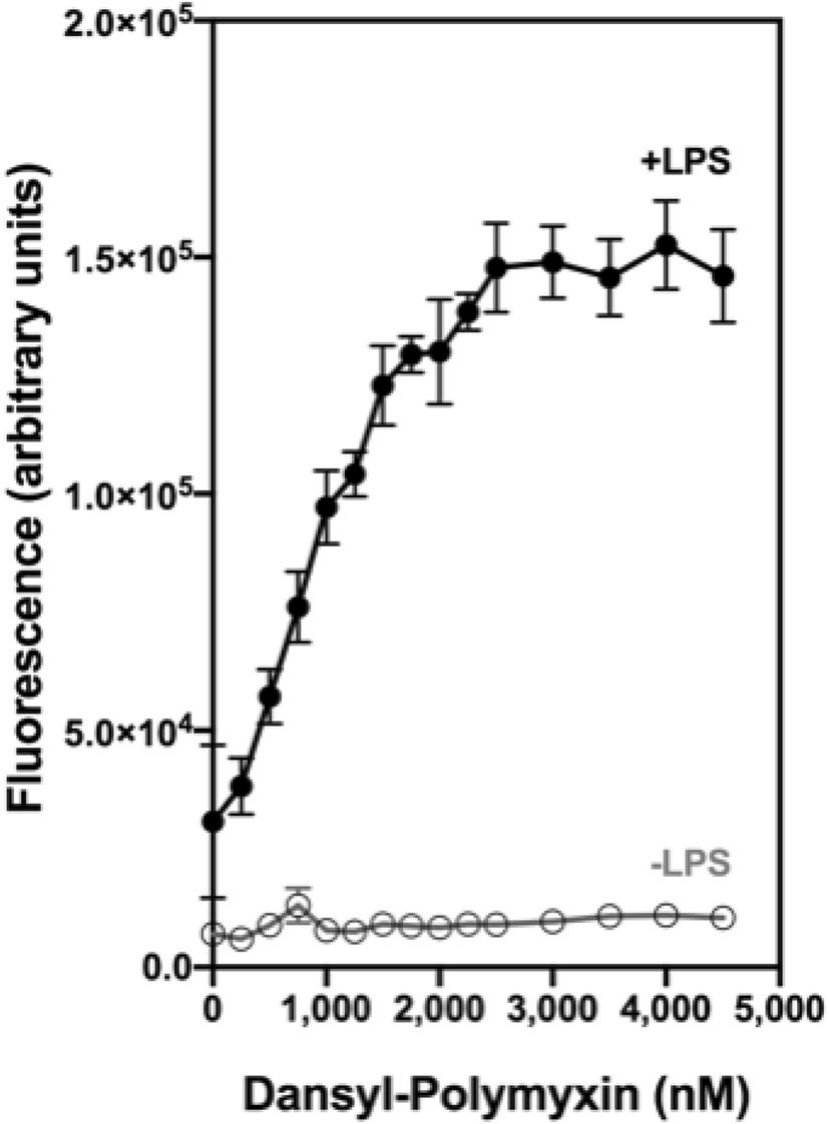
Fluorescence of DPX upon binding LPS. DPX was added in 0.25 μM increments to a cuvette containing either 3 μg/mL LPS (●), or buffer alone (○). LPS becomes saturated by DPX when approximately 2.5 μM DPX is added in solution, as denoted by a plateau in rising fluorescence. Data points represent the average from n = 3 independent experiments; error bars depict the standard error of the mean.

**Figure 3:**
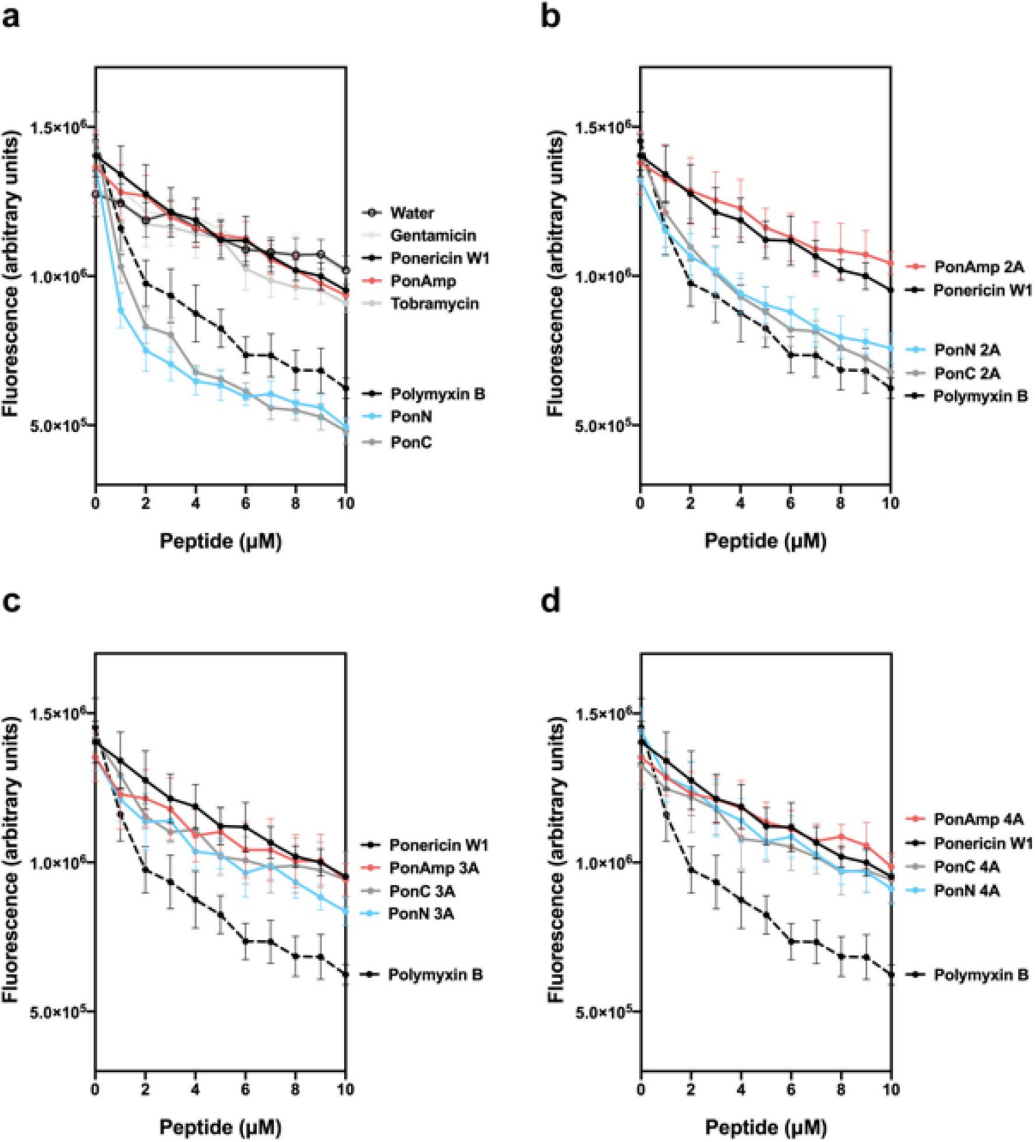
Displacement of DPX from LPS by (**a**) Native Ponericin W1 and parent peptides; (**b**) 2A analogs; (**c**) 3A analogs; and (**d**) 4A analogs. Increasing concentrations of peptide were titrated into a cuvette containing 3 μg/mL LPS saturated by 2.5 μM DPX. As depicted along with the parent peptides, native Polymyxin B (i.e., no dansyl group attached) was used as a positive control for displacement. Water, in addition to two small molecule antibiotics with intracellular targets (gentamicin and tobramycin) were used as negative controls. The legend to the right of each figure is labelled from top to bottom in order of increasing DPX displacement affinity. Data points represent the average from n = 3 independent experiments; error bars depict the standard error of the mean.

We then sought to evaluate whether the relative trends in antimicrobial efficacy observed between Lys-clustered versus helical amphipathic sequences against gram-negative bacteria correlate to their degree of interaction with the OM. In doing so, we tested the binding affinity of our peptide library to LPS using a Dansyl-Polymyxin (DPX) displacement assay^46^. DPX is a fluorescently-tagged derivative of Polymyxin B that highly associates with polyanionic sites on LPS^46^. Dansyl fluorescence reaches a maximum when surrounded by a hydrophobic environment (ex. lipid A moiety of LPS), thereby enabling us to track bound and unbound states of the LPS-DPX complex. First, we empirically determined the concentration of DPX required to completely saturate a set quantity of LPS in solution (Fig. 2). Upon identifying the saturation threshold, we assessed the ability of our peptide library to outcompete for LPS binding by displacing bound DPX. Lys-clustered PonN and PonC were found to highly interrupt the LPS-DPX complex, as denoted by sharp drops in fluorescence with each addition of peptide (Fig. 3a). Comparatively, a negligible change in fluorescence is seen for Ponericin W1 and PonAmp, even at the highest tested concentration. High sequence hydrophobicity is observed to supplement peptide propensity to displace DPX as the binding affinity of PonN and PonC to LPS progressively weakens with increasing Leu-to-Ala substitutions (Fig. 3b–d).

### LPS induces helical formation in peptides

**Figure 4:**
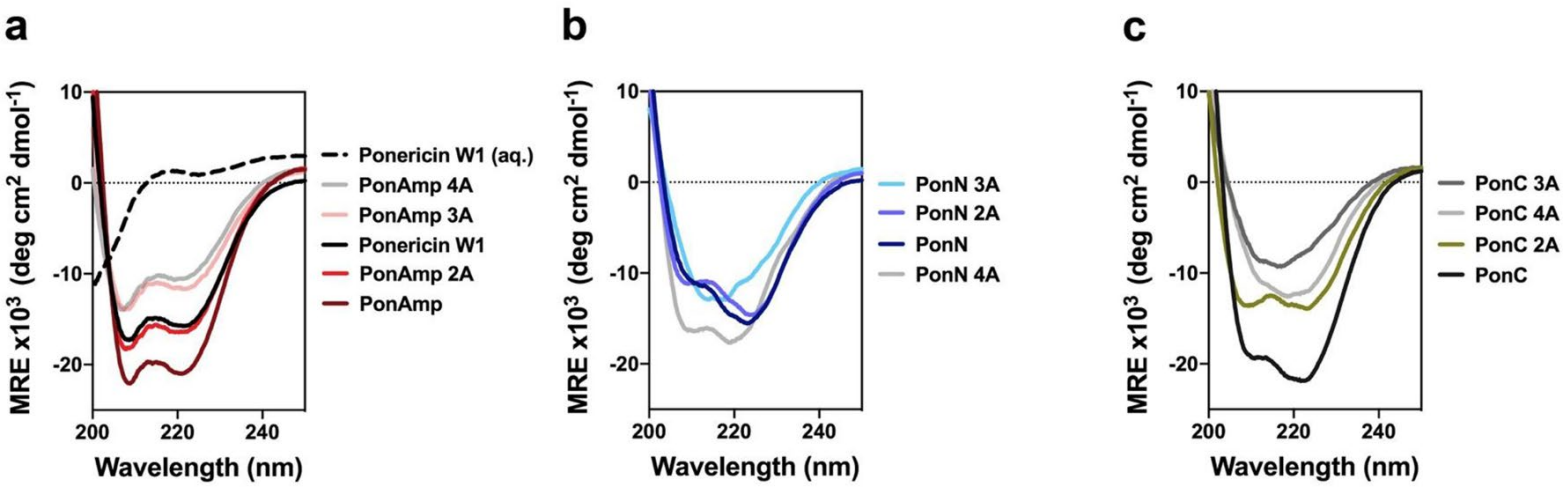
CD spectra of (**a**) PonAmp peptides; (**b**) PonN peptides; and (**c**) PonC peptides. 25 μM peptide was incubated with 2 mg/mL of LPS overnight to achieve equilibrium. Spectra for native Ponericin W1 in the presence of LPS (), as well as in aqueous solution alone (), are shown in (**a**). The legend to the right of each figure is labelled from top to bottom in order of increasing helicity. Spectra represent the average from n = 3 independent experiments.

While CAPs are capable of adopting a range of secondary structures at the IM, many form an α-helix as they partition into the membrane from an otherwise randomly coiled conformation in aqueous solution^1^. However, the initial structuring of CAPs at the OM, and whether this impacts peptide efficacy in proceeding steps, remain unclear. As such, we performed circular dichroism (CD) spectroscopy to elucidate the nature of peptide folding in the presence of LPS micelles. In aqueous solutions, LPS spontaneously organize into micelles above a critical threshold concentration, thereby enabling us to work with the basic architecture of the OM in vitro^47^. Our CD spectra delineate helical folding in native Ponericin W1 and across the PonAmp series (Fig. 4a) while Lys-clustered PonN and PonC peptides initially retain helical character but gradually demonstrate a loss of this propensity with increasing Leu-to-Ala content (Fig. 4b,c), in effect, displaying spectra that more closely resemble a random coil.

### Peptide embedment into LPS micelles

Upon confirming the binding potential and folding of our peptide library at the LPS interface, we undertook to deduce whether peptides are capable of inserting into the hydrophobic milieu of the LPS lipid core. All sequences in our peptide library contain a Trp residue which was used as a natural fluorescent probe of environment polarity. A characteristic blue shift from 350 nm (empirically determined baseline value reflecting the fluorescence of Trp in aqueous solution alone) is observed for all peptides in the presence of LPS, with PonN exhibiting the most pronounced change (326 nm; Table 2). In agreement with earlier findings, reductions to sequence hydrophobicity are then seen to result in comparatively less stable residence of peptides within the LPS micelle, as indicated by maximum fluorescence wavelength values (λ_MF_) that gradually recede back to baseline with increasing Leu-to-Ala substitutions (Table 2).

**Table 2:** Blue shifts in Trp fluorescence (λ_MF_) and Stern–Volmer slopes for acrylamide quenching in the presence of LPS micelles (*m*). ^a^ Trp fluorescence of the peptide library was measured from 300 to 400 nm. Summarized data indicate the wavelength corresponding to peak fluorescence. Values closer to 300 nm denote a greater blue shift, broadly corresponding to greater interactions between peptide and the hydrophobic lipid region of LPS micelles.

Peptide	λ_MF_ (nm) ^a^	*m* (F_0_/F) ^b^
Ponericin W1	338	5.9
PonAmp	334	4.7
PonAmp 2A	336	3.8
PonAmp 3A	336	4.5
PonAmp 4A	336	3.9
PonN	326	3.4
PonN 2A	330	3.2
PonN 3A	340	4.0
PonN 4A	336	3.7
PonC	335	3.8
PonC 2A	346	5.8
PonC 3A	346	5.6
PonC 4A	346	5.8

^b^ Stern–Volmer slopes reflecting area under the curve (AUC) for Trp fluorescence measured from 310 to 400 nm following titrations of a water-soluble quencher. Values closer to 1.0 denote less exposure to acrylamide and greater peptide insertion into the LPS micelle. Data represent the average from n = 3 independent experiments; standard error of the mean is shown for parent peptides in Fig. 5.

**Figure 5:**
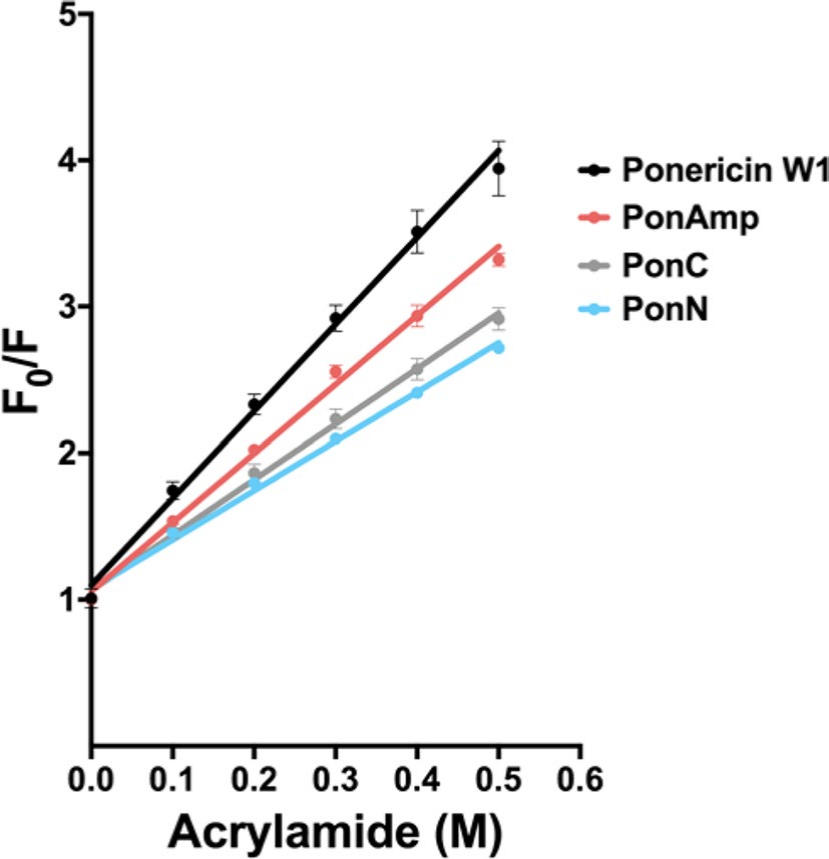
Stern–Volmer quenching slopes of native Ponericin W1 and Parent peptides. 10 μM peptide was incubated with 0.8 mg/mL of LPS overnight to achieve equilibrium. The legend to the right is labelled from top to bottom in order of decreasing steepness of Stern–Volmer slopes. Data represent the average from n = 3 independent experiments; error bars depict the standard error of the mean.

Addition of acrylamide, a water-soluble quencher of Trp fluorescence, reveals the relative embedment and shielded nature of Trp residues in all peptide analogs within the hydrophobic space of LPS micelles (Table 2, Fig. 5). PonN and PonC display shallow Stern–Volmer slopes (F_0_/F; *m* = 3.4 and 3.8 respectively) relative to Ponericin W1 (*m* = 5.9) and PonAmp (*m* = 4.7), implying deeper integration of Lys-clustered sequences into the lipid core of LPS micelles compared to helical amphipathic counterparts (Fig. 5). Increased Leu-to-Ala substitutions are generally seen to compromise the degree of peptide insertion into LPS micelles (Table 2).

## Discussion

The bacterial OM is the primary barrier of an intact gram-negative bacterial cell and often the principal mechanistic target for resistance to CAPs^48–50^. A prevailing model of how CAPs navigate across this initial barrier involves the concept of self-promoted uptake. In this model of permeation, CAPs bind to LPS along the outer leaflet of the OM by virtue of strong electrostatic interactions with the anionic phosphate groups that line the lipid A base^51–57^. This action displaces divalent cations (Mg^2+^ and Ca^2+^) that normally cross-bridge the space between adjacent LPS molecules, resulting in local disruptions along the LPS continuum and enabling peptide entry^53,54^. The significance of likewise OM interactions has been reported to substantially influence overall bacterial susceptibility to peptide activity^58–61^.

The present study provides understanding of the peptide-OM dynamic in native Ponericin W1 by showing that modifications to the primary peptide sequence directly impact the propensity of its designed derivatives to engage with LPS. Importantly, our results highlight the advantage of congregating Lys residues. As shown in Fig. 3, Lys-clustered PonN and PonC highly inhibit the LPS-DPX complex by outcompeting for anionic binding sites, thereby indicating their enhanced chemical compatibility with the OM interface. The preferential engagement of Lys-clustered sequences with LPS is likely due to enrichment in the perceived local density of positive character endowed by the uninterrupted chain of charged residues at the peptide terminus. While molecules hosting greater net-positive charge are generally reported to exhibit higher binding affinity to LPS^46^, to our knowledge this is the first account to recognize the importance of charged residue placement in this interaction.

Hydrophobicity modified analogs of PonN and PonC were also seen to sustain high levels of antimicrobial activity against the gram-negative bacterium *P. aeruginosa* relative to their helical amphipathic counterparts, while analogs of PonAmp better retained activity against the gram-positive *B. subtilis*, which lack an OM. These interpretations are consistent with literature underscoring the importance of OM interactions in predicting overall antimicrobial function against gram-negative bacteria^62^. The observed variance in peptide activity between the two model organisms points toward two key observations: (1) Lys-clustered PonN and PonC display a heightened advantage for activity at the OM, while (2) a helical amphipathic sequence such as PonAmp is preferred for activity at the IM. An explanation for this apparent dichotomy can be attributed to the distinct composition of the outer and inner bacterial membranes. Thus, the first line of contact at the OM involves LPS, which comprises approximately 75% of the outer leaflet^27^. As seen from CD (Fig. 4), the majority of sequences in our peptide library adopt secondary structure in the presence of LPS micelles from an initial unstructured form in aqueous solution. Notably, peptides featuring reduced hydrophobicity are correspondingly less capable of spontaneous partitioning into hydrophobic membrane environments and instead display spectra likely reflecting a hybrid population comprised of peptides that adopt helices (membrane-interacting) and those that remain unstructured (viz., solvated in the aqueous phase). In general, the electrostatic traction of Lys-clustered peptides to LPS, in combination with their structural propensity, likely function in synergy to enhance peptide uptake across the OM.

In contrast, Lys-clustered PonN and PonC have been previously found to exclusively adopt random coils when exposed to liposomes composed of an approximate 3:1 ratio of PE:PG phospholipids, representative of the bacterial IM^37^. The latter configuration ostensibly deters peptide capacity for pore formation and/or carpeted assembly compared to helical amphipathic constructs such as PonAmp, which continues to display high helical propensity at both membrane interfaces^37^. The comparatively shallow and/or transient nature of interaction between Lys-clustered constructs and the IM interface was further determined using an in vitro dye release assay where liposome mimetics of the bacterial IM ruptured instantaneously when exposed to PonAmp but more gradually, with a slower disruption trajectory in the presence of either PonN or PonC^37^.

At the IM, high hydrophobicity is another fundamental property of CAPs that governs peptide activity^42^. Considerations of hydrophobicity are critical when designing CAPs with balanced antimicrobial efficacy and low mammalian toxicity as high sequence hydrophobicity often promotes off-target interactions with mammalian membranes^42^; previous work on the Ponericin W1 library revealed that a minimum of two Leu-to-Ala substitutions effectively removes peptide toxicity against human red blood cells^37^. These earlier findings reinforce the notion that distinct hydrophobicity thresholds are required for spontaneous membrane insertion contingent on bilayer composition. Thus, in conjunction with systematic studies on charged residue placement, here we sought to explore whether hydrophobicity levels similarly influence peptide behavior at the OM level. We found that peptides hosting dampened sequence hydrophobicity generally bound to LPS with decreasing affinity and were less stably embedded within the micellar environment. This trend was apparently independent of Lys placement, but was evident across our Lys-clustered sequences, which displayed a clear functional advantage over amphipathic designs at high hydrophobicity.

**Figure 6:**
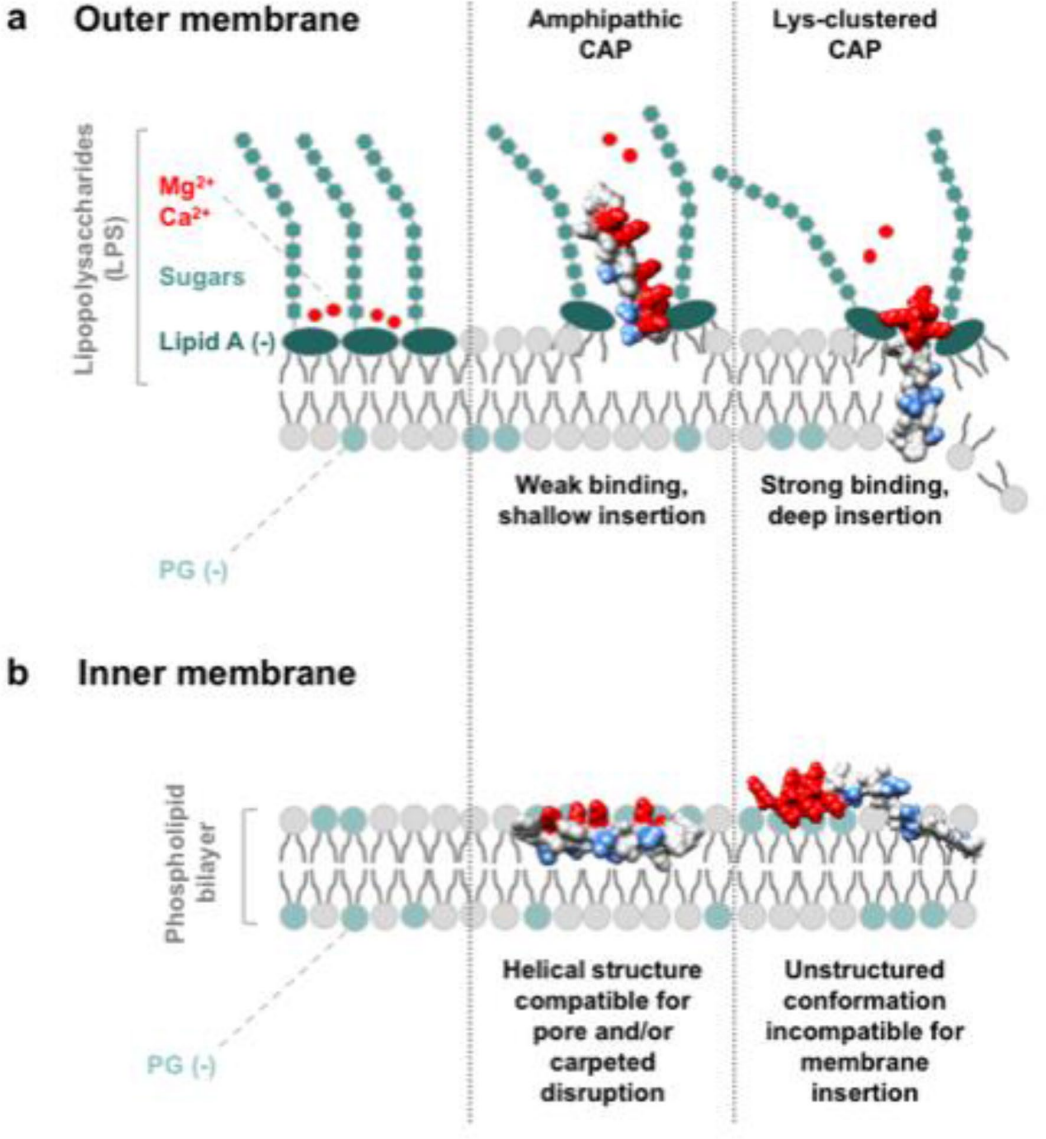
Schematic representation of a proposed mechanism of action for amphipathic and Lys-clustered peptides at the bacterial (**a**) OM, and (**b**) IM. Lys residues are rendered in red along the peptide structure; sites for Leu-to-Ala substitutions are rendered in blue. Helical amphipathic sequences are modelled to adopt helical conformations at both the OM and IM, with preferential activity at the latter interface via a positively charged face that electrostatically binds to anionic PG head groups, and a hydrophobic face that penetrates into the hydrophobic bilayer core. Lys-clustered sequences are shown to retain helical character exclusively at the OM, where congregation of charged residues facilitates strong binding affinity to the lipid A component of LPS; the uninterrupted hydrophobic chain then deeply inserts into the lipid core.

Figure 6 schematically summarizes the present work, where we designed sets of Lys-clustered and helical amphipathic peptides—each with a range of hydrophobicities—to evaluate the different modes of peptide interactions at the outer versus inner bacterial membranes. Taken together, we propose that the systematic rearrangement of charged residues within the primary sequence of Ponericin W1 can be implemented as a means of targeting peptide activity towards either gram-negative or gram-positive species. A study by Gong et al.^63^ on the influence of net charge and hydrophobicity on peptide-LPS interactions using solid-state nuclear magnetic resonance measurements reached a converging conclusion to the work presented herein.

Our results offer a preliminary explanation for the rare occurrence of charge clustering in nature (< 2% of natural CAPs contain a cluster of four or more charged residues^64^) which may relate to the evolutionary development of CAPs with high affinity for amphipathic secondary structures against the IM of gram-positive bacteria, and thus suggest that Lys-clustering can therefore be applied as a promising design parameter when synthesizing de novo CAPs with enhanced potency against gram-negative species. This is particularly relevant in scenarios where the native expression of OM porins required for drug entry is limited, as seen in *P. aeruginosa*^65^. Moreover, the sustained antimicrobial efficacy of Lys-clustered peptides against MDR *P. aeruginosa—*up to four Leu-to-Ala substitutions—underscores a pivotal advantage that broadens the therapeutic index of CAPs for human use.

We have shown here that Ponericin-based peptides hosting N- or C-terminally clustered Lys residues exhibit enhanced chemical compatibility with LPS, and thus constitute a feature that can be utilized to enhance uptake of the proposed peptide library across the OM of gram-negative bacteria. The high affinity of PonN and PonC to LPS afforded by Lys clustering endows an apparent advantage for antimicrobial activity against MDR *P. aeruginosa,* even within the context of reduced sequence hydrophobicity. As such, we have been able to demonstrate the design of a set of peptides derived from natural Ponericin W1 that balances efficacy and safety towards mammalian host cells. In particular, we note that within our designed library, Lys-clustered PonN 2A appears to be a lead construct that retains high LPS binding and insertion parameters for bacterial OM permeation while working within the hydrophobicity limits of a sequence that is safe towards mammalian cells.

## Methods

### Bacterial isolates

Clinical isolates of *P*. *aeruginosa* [Research Ethics Board (REB) #1000019444] were obtained with informed consent from CF patients with chronic infection followed at the Hospital for Sick Children (Toronto, Canada)^66^. Consent was obtained from a parent or legal guardian if not of age. All methods were performed in accordance with the relevant guidelines and regulations for research involving human subjects at the Hospital for Sick Children. Antimicrobial susceptibility testing was performed as per the Clinical Laboratory Standards Institute (CLSI)^67^.

### Peptide quantification

Peptides were purchased from Biosynth (formerly Vivitide) with ≥ 95% purity. Peptides were received as lyophilized powders, which were resuspended in water and measured for absorbance at 280 nm. Peptide concentration was determined at an extinction coefficient of 5690 M^−1^ cm^−1^ and samples were kept as frozen stocks until use.

### Minimum inhibitory concentration (MIC) assay

Three clinical isolates of MDR *P. aeruginosa* were previously obtained from CF patients with chronic infection at the Hospital for Sick Children [Research Ethics Board (REB) #1000019444] (Toronto, Canada) and selected for use in this work along with a laboratory strain of *B. subtilis*^66^. Serial dilutions of peptides (10 μL) were prepared to achieve concentrations from 50 to 0 μM on a sterile 96-well clear bottom plate. Cells were resuspended in Mueller − Hinton Broth (MHB) and added at 10^5^ CFU/well to achieve a final volume of 100 μL. Peptide and bacteria were allowed to incubate at 37 °C for 20 h. OD_600_ was measured at the end of the incubation period. Reports of MIC are background subtracted from an MHB-only control.

### Dansyl-polymyxin (DPX) displacement assay

Lyophilized stocks of LPS from *P. aeruginosa* 10 (#L9143) and Dansyl-labelled polymyxin B (DPX) suspended in water (#SBR00029) were purchased from Sigma. 1 mg/mL LPS was prepared in water and stored at 4 °C until use. As described in detail elsewhere^46^, saturation of the LPS-DPX complex was first determined on a Photon Technology International fluorometer using a 1 cm cuvette. Stepwise additions of DPX (in 0.25 μM increments up to 5 μM) was added to 3 μg/mL LPS suspended in 10 mM Tris 10 mM NaCl buffer at pH 7.4. DPX fluorescence was excited at 340 nm (2 nm slit width) and emission was recorded at 485 nm (5 nm slit width). Saturation of LPS was reached at approximately 2.5 μM DPX, as determined by a plateau in rising DPX fluorescence, consistent with other work^46^. Additions of DPX to buffer in the absence of LPS showed no changes in fluorescence levels to provide a background reading.

To measure DPX displacement, peptides were independently added at 1 μM increments (up to 10 μM) to preparations containing 3 μg/mL LPS saturated by 2.5 μM DPX. DPX fluorescence was read immediately upon peptide addition. Drops in fluorescence are interpreted as the removal of DPX from LPS due to competitive binding by peptide.

### Circular dichroism (CD) spectroscopy

CD spectra were obtained on a Jasco J-720 spectropolarimeter using a 0.1 cm cuvette with three accumulations recorded per run. CD samples were suspended in 10 mM Tris 10 mM NaCl buffer at pH 7.4 containing 25 μM peptide and 2 mg/mL LPS. LPS spontaneously form micelles above a critical concentration of approximately 14 μg/mL in any solution^47^. Samples were allowed to equilibrate overnight prior to reading. Final spectra represented the average of three independent replicates corrected for background noise and are presented as mean residue molar ellipticity (MRE) calculated using standard formulas.

### Tryptophan fluorescence and quenching

Trp fluorescence was measured on a Photon Technology International fluorometer using a 1 cm cuvette. Samples were suspended in 10 mM Tris 10 mM NaCl buffer at pH 7.4 containing 10 μM peptide and 0.8 mg/mL LPS and allowed to equilibrate overnight prior to reading. Trp was excited at 280 nm (2 nm slit width) and emission spectra were recorded from 300 to 400 nm (5 nm slit width) with a step size of 2 nm.

For quenching experiments, acrylamide was added in 0.1 M increments to the above preparation containing 10 μM peptide and 0.8 mg/mL LPS. To avoid overlapping signals from Trp and acrylamide, Trp was instead excited at 295 nm and emission spectra were recorded from 310 to 400 nm with a step size of 2 nm. Stern–Volmer plots were constructed by plotting F_0_/F against increasing acrylamide concentration. Steeper slopes denote greater quenching and are interpreted as Trp exposed to the surrounding aqueous solution.

## Data Availability

All data generated or analyzed during this study are included in this article.
